# Effects of the visual environment on object localization in posterior cortical atrophy and typical Alzheimer's disease

**DOI:** 10.3389/fmed.2023.1102510

**Published:** 2023-02-28

**Authors:** Dilek Ocal, Ian D. McCarthy, Teresa Poole, Silvia Primativo, Tatsuto Suzuki, Nick Tyler, Chris Frost, Sebastian J. Crutch, Keir X. X. Yong

**Affiliations:** ^1^Dementia Research Centre, Department of Neurodegeneration, UCL Institute of Neurology, University College London, London, United Kingdom; ^2^Pedestrian Accessibility and Movement Environment Laboratory, Department of Civil, Environmental and Geomatic Engineering, Faculty of Engineering Science, University College London, London, United Kingdom; ^3^Department of Medical Statistics, Faculty of Epidemiology and Population Health, London School of Hygiene and Tropical Medicine, London, United Kingdom

**Keywords:** posterior cortical atrophy, object localization, dementia, vision, clutter, reaching, environmental modifications, Alzheimer's disease

## Abstract

**Introduction:**

Visual processing deficits in Alzheimer's disease are associated with diminished functional independence. While environmental adaptations have been proposed to promote independence, recent guidance gives limited consideration to such deficits and offers conflicting recommendations for people with dementia. We evaluated the effects of clutter and color contrasts on performances of everyday actions in posterior cortical atrophy and memory-led typical Alzheimer's disease.

**Methods:**

15 patients with posterior cortical atrophy, 11 with typical Alzheimer's disease and 16 healthy controls were asked to pick up a visible target object as part of two pilot repeated-measures investigations from a standing or seated position. Participants picked up the target within a controlled real-world setting under varying environmental conditions: with/without clutter, with/without color contrast cue and far/near target position. Task completion time was recorded using a target-mounted inertial measurement unit.

**Results:**

Across both experiments, difficulties locating a target object were apparent through patient groups taking an estimated 50–90% longer to pick up targets relative to controls. There was no evidence of effects of color contrast when locating objects from standing/seated positions and of any other environmental conditions from a standing position on completion time in any participant group. Locating objects, surrounded by five distractors rather than none, from a seated position was associated with a disproportionately greater effect on completion times in the posterior cortical atrophy group relative to the control or typical Alzheimer's disease groups. Smaller, not statistically significant but directionally consistent, ratios of relative effects were seen for two distractors compared with none.

**Discussion:**

Findings are consistent with inefficient object localization in posterior cortical atrophy relative to typical Alzheimer's disease and control groups, particularly with targets presented within reaching distance among visual clutter. Findings may carry implications for considering the adverse effects of visual clutter in developing and implementing environmental modifications to promote functional independence in Alzheimer's disease.

## 1. Introduction

Alzheimer's disease (AD), the most common form of dementia, is characterized by an insidious deterioration of multiple cognitive domains, including memory, language, executive function and visual processing. This deterioration is accompanied by a progressively diminishing capacity to carry out everyday activities independently (1, 2) which in turn is associated with institutionalization, increased carer burden and decreased quality of life for those living with the disease (3, 4). Visual processing deficits are common yet under-recognized consequences of AD and have been strongly associated with diminished autonomy, indeed more so than memory (5, 6).

Corticovisual dysfunction is a core feature of posterior cortical atrophy (PCA), a neurodegenerative syndrome most commonly associated with AD pathology (7, 8), which is characterized by a progressive decline in visuoperceptual and visuospatial abilities (9) and posterior parietal, occipital, and occipito-temporal atrophy (10, 11). In contrast to the predominantly memory-led presentation of typical AD (tAD), patients with PCA often demonstrate a variety of visual and posterior impairments while maintaining relatively spared episodic memory, language, executive functions and behavior (9, 12, 13); by comparison, patients with tAD exhibit deficits in visual function at later disease stages (14, 15). Examples of deficient visual processing in PCA and tAD include impairments in visual search behaviors (16–18), perceiving objects surrounded by visual clutter (2, 19, 20) and executing goal-directed reaching due to difficulties in localizing objects in relation to oneself (21, 22).

The physical environment may play an important role in managing the challenges that patients with PCA and tAD experience with everyday activities. For example, Dunne et al. (23) provided evidence that using high-contrast tableware increased liquid and food intake in patients with advanced tAD. Similarly, color and contrast adaptations are commonly cited as important approaches to supporting effective localization of signs, toilets and handrails in patients with tAD (24, 25), and we recently provided evidence of color contrast-based cues supporting navigation to destinations in a combined group of PCA and tAD patients (26). These findings invite the exploration of perceptual conditions that may support patients to carry out everyday actions such as localizing and reaching for objects. However, the evidence base for environmental guidance to support independence in dementia has been noted as weak or contentious (27–29). Of particular relevance to the current study are questions regarding whether the introduction of “landmarks” or objects intending to promote navigation, or to support reminiscence, in practice has adverse effects as “clutter.” Notably, PCA patients commonly exhibit particular difficulties perceiving objects presented among visual clutter (19, 30), and Giovannetti et al. (1, 20) reported that patients with all-cause dementia made more object localization errors in the presence of clutter, especially when target and clutter were visually similar. This has prompted calls for more empirical research (27, 31).

The aim of the current study was to investigate the effects of visual clutter and color-contrast cues on object localization performance in patients with PCA and tAD, relative to healthy controls. Participants were asked to pick up a target object during two experiments (reaching from different standing positions; reaching from a seated position) conducted within a controlled real-world setting. The target was presented under varying conditions of clutter and position, with or without a color contrast cue. Our main hypotheses were that minimizing clutter and introducing a color contrast cue would reduce the time taken to pick up the target in both patient groups. A subsidiary hypothesis was that object localization deficits would be more apparent in PCA relative to tAD owing to the greater extent of corticovisual impairment.

## 2. Materials and methods

### 2.1. Participants

A total of 16 healthy controls, 15 PCA and 11 tAD patients took part in one or two experiments aimed at assessing the impact of visual clutter and color-contrast cues on object localization skills. PCA patients fulfilled consensus diagnostic criteria for PCA-pure (32) and tAD patients fulfilled research criteria for probable AD (33). Patients were recruited at the Dementia Research Centre and the National Hospital for Neurology and Neurosurgery London. Controls were recruited from a local database and did not have a history of neurological or psychiatric illness. Ethical approval for the study was provided by the National Research Ethics Service Committee London Queen Square and informed consent was obtained from all participants. Molecular pathology was available for 6/11 tAD patients and 7/15 PCA; all were consistent with AD pathology (positive amyloid scan on standard visual rating or CSF Aβ1-42 ≤ 450 and/or tau/Aβ ratio > 1). Both patient groups underwent a battery of neuropsychological testing assessing general cognitive ability, early visual/visuoperceptual/visuospatial processing and verbal/non-verbal memory. See Tables 1, 2 for participant demographics and details of the neuropsychology assessments, respectively.

**Table 1:** Demographic characteristics of participant groups.

	**Control**			**PCA**			**tAD**			
	**#**	**M**	±**SD**	**#**	**M**	±**SD**	**#**	**M**	±**SD**	
**Experiment 1**										
*N*	16	−	−	15	−	−	11	−	−	
Sex (F:M)	9:7	−	−	8:7	−	−	4:7	−	−	
Age	−	67.0	± 6.4		68.7	± 6.3		68.9	± 6.5	
MMSE (/30)	−	−	−	−	20.5	± 5.3	−	20.9	± 6.0	
β-Amyloid PET/CSF consistent with AD^*^	−	−	−	7/7	−	−	6/6	−	−	
**Experiment 2**										
*N*	14	−	−	7	−	−	6	−	−	
Sex (F:M)	7:7	−	−	5:2	−	−	4:2	−	−	
Age	−	67.0	± 6.3		69.0	± 9.3		66.0	± 2.7	
MMSE (/30)	−	−	−	−	17.6	± 3.6	−	21.7	± 6.5	
β-Amyloid PET/CSF consistent with AD^*^	−	−	−	4/4	−	−	3/3	−	−	

Presented are number of participants (N), mean (M) and standard deviation (± SD) for experiment 1 and experiment 2. #: number or ratio;^*^positive amyloid imaging performed as part of another investigation or CSF Aβ1-42 ≤ 627 and/or tau/Aβ ratio ≥ 0.52. MMSE, Mini Mental State Examination (34) (maximum score shown in parenthesis); PCA, posterior cortical atrophy; tAD, typical Alzheimer's disease.

**Table 2:** Medians, interquartile ranges of neuropsychological scores and estimated performance relative to normative datasets for patient groups.

		**PCA**		**tAD**		**Below 5th %ile**	
	**Max**	**Mdn**	**Q1–Q3**	**Mdn**	**Q1–Q3**	**PCA**	**tAD**
**Background psychology**							
SRMT^a^ (words)	25	21.0	19.3−23.0	18.0	15.3−18.0	2/14	7/10
SRMT (faces)	25	18.0	17.0−21.0	20.5	18.3−21.8	4/15	2/10
Concrete synonyms^b^	25	20.0	18.5−23.5	20.0	18.0−23.5	4/15	3/11
Naming (verbal description)	20	14.0	10.5−19.0	17.0	13.0−18.0	10/15	5/11
Cognitive estimates^c^	0	10.5	7.0−14.8	9.0	6.5−15.5	11/14	8/11
Calculation (GDA)^d^	24	1.0	0.0−3.0	6.0	0.5−13.0	8/11	4/10
Spelling (GDST)^e^	20	7.5	3.8−13.5	14.0	6.5−19.0	4/14	1/11
Reading (CORVIST)^f^	16	16.0	14.0−16.0	16.0	16.0−16.0	−	−
Gesture production	15	13.0	11.0−14.0	15.0	12.5−15.0	−	−
Digit span—forwards	12	5.0	4.0−6.0	7.0	5.5−8.0	5/15	3/11
Digit span—max forwards	8	5.0	4.0−5.0	6.0	5.5−7.0	−	−
Digit span—backwards	12	3.5	3.0−5.0	4.0	3.0−5.0	2/14	2/11
Digit span—max backwards	7	3.0	3.0−3.6	3.0	4.0−1.0	−	−
**Early visual processing**							
Figure-ground discrimination^g^	20	17.0	13.3−19.0	19.0	18.5−20.0	8/12	3/11
Shape discrimination (B1)^h^	20	15.0	13.0−18.0	20.0	19.5−20.0	−	−
Hue discrimination (CORVIST)	4	2.5	1.0−3.0	4.0	3.0−4.0	−	−
Crowding^i^	10	9.0	6.0−10.0	10.0	10.0−10.0	−	−
Visual acuity (CORVIST)	6/9	6/9	−	6/9	−	−	−
**Visuoperceptual processing**							
Fragmented letters (VOSP)^g^	20	2.0	0.0−10.5	19.0	17.3−19.8	11/11	2/10
Object decision (VOSP)	20	11.0	8.0−16.0	17.0	16.5−18.5	8/13	1/11
**Visuospatial processing**							
Number location (VOSP)	10	4.5	0.8−6.8	9.0	2.5−9.0	7/10	5/11
Dot counting (VOSP)	10	6.0	4.3−8.5	10.0	8.5−10.0	9/12	3/11
A-Cancellation (time)^j^	90s	90s	70.0−90.0	39.0s	26.8−42.6	13/14	7/11
A-Cancellation (items)^k^	19	10.0	1.1−13.5	0.0	0.0−0.5	−	−

Mdn, median; Q1–Q3: interquartile ranges; PCA, posterior cortical atrophy; tAD, typical Alzheimer's disease.^a^Short recognition memory test—joint auditory/visual presentation (35).^b^Concrete and abstract word synonym test (36).^c^General knowledge-based questions (37).^d^Graded difficulty arithmetic test (38).^e^Graded difficulty spelling test—set B first 20 items (39).^f^Cortical visual screening test (40).^g^VOSP, Visual Object and Space Perception Battery (41).^h^Oblong edge ratio 1:1.20 (42).^i^10 alphanumeric strings.^j^Completion time (43).^k^Number of items missed (43).

### 2.2. Experimental setting

**Figure 1:**
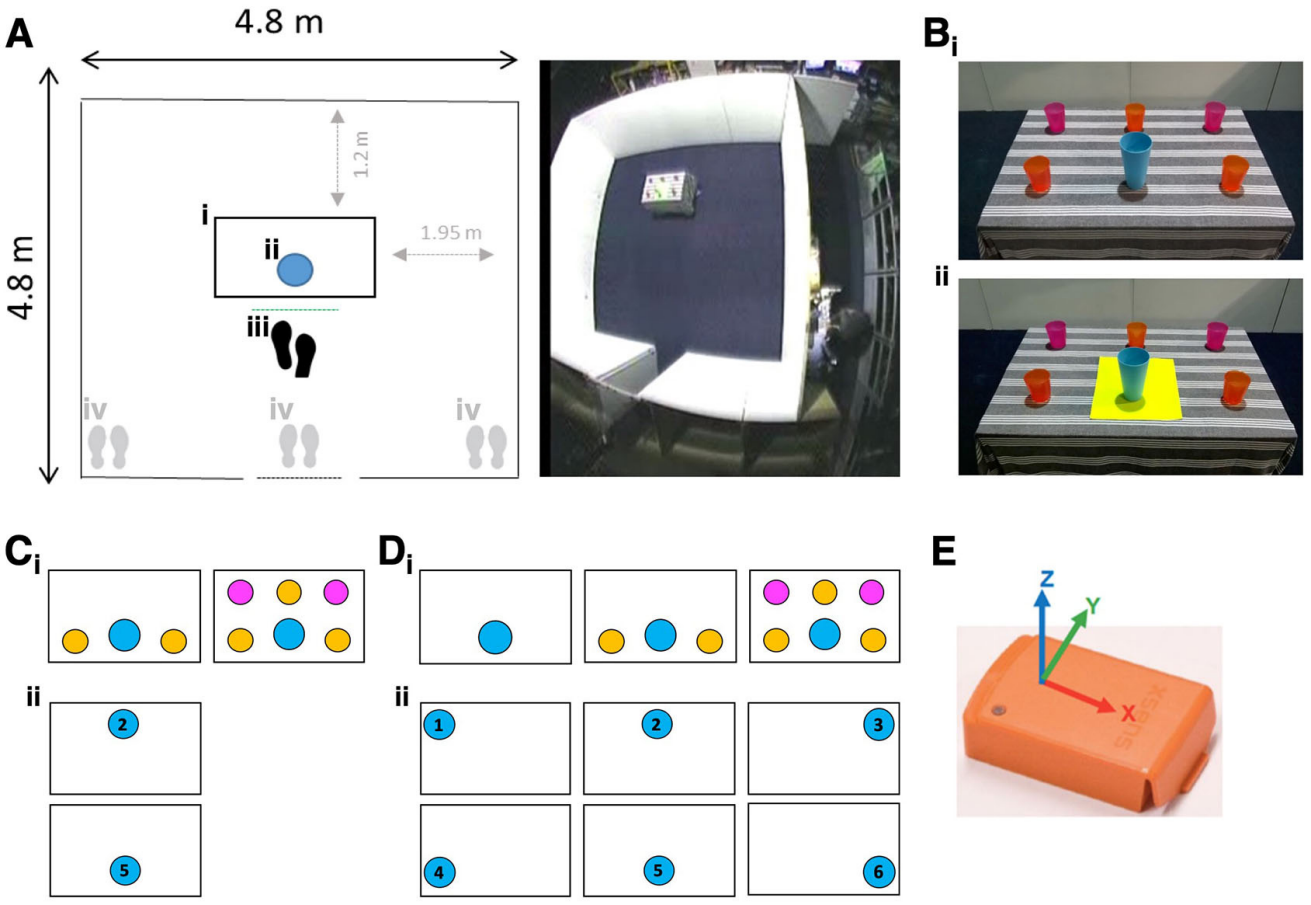
**(Ai)** Table position, **(Aii)** target position, **(Aiii)** starting position from reaching distance (proximal), **(Aiv)** starting position from far left, right, or center of the setting. **(Bi)** Blue target cup surrounded by clutter, **(Bii)** blue target cup with yellow color-contrast cue. **(Ci)** Clutter conditions (2 vs. 5 distractors) for Experiment 1, **(Cii)** Target positions for Experiment 1. **(Di)** Clutter conditions for Experiment 2 (2 vs. 0; 5 vs. 0 distractors), **(Dii)** target positions for Experiment 2. **(E)** Inertial measurement unit (IMU - Xsens MT, image courtesy of Xsens) mounted within the base of the target object.

The experimental setting was constructed at the Pedestrian Accessibility Movement and Environment Laboratory (PAMELA) at UCL. The setting consisted of an open room [4.8 m (D) × 4.8 m (W) × 2.0 m (H)] with an entry corridor and a table [60 cm (D) × 90 cm (W) × 74 cm (H)] on which both target object and distractor objects (clutter) were placed (Figure 1A). The target was a blue cup [94 mm (top outside diameter) × 56 mm (bottom outside diameter) × 155 mm (H)]; distractors were cups differing in size and color from the target (Figure 1B).

#### 2.2.1. Experiment 1: Object localization from standing positions

##### 2.2.1.1. Environmental conditions

Participants were standing in front of the table and asked to pick up the target object under the following environmental conditions:

1. Clutter: The target was presented among either 2 or 5 distractors (Figure 1Ci).
2. Cue: The target was presented either with or without a color contrast visual cue (21 cm × 30 cm yellow placemat) (Figure 1B).
3. Starting position: Participants were asked to pick up the target from one of four starting standing positions in front of the table: within reaching distance of the target (proximal—Figure 1Aiii), or approaching the target from a distance (from far left, right, or center of the setting; Figure 1Aiv).
4. Target position: The target was positioned on the near or far side of the table relative to starting standing positions (Figure 1Cii).

##### 2.2.1.2. Procedure

Participants underwent two practice trials during which their dominant hand preference was determined. An Arduino-based timing system fulfilled the following functions: playing an audio signal indicating the start of each trial and recording the start of each trial at 1,000 Hz. Between each trial, participants' view of the setting was obscured by an occluding screen and participants were instructed to keep their hands by their sides. Trials were administered through a repeated-measures design such that each participant performed 32 trials, one for each combination of clutter (2 or 5 distractor cups); cue (not present/present); target position (near/far) and starting position (proximal/left/right/center). To control for order effects cue and clutter variables were arranged in four counterbalanced variants of a Latin square design with variants randomly assigned to each participant.

#### 2.2.2. Experiment 2: Object localization from a seated position

A subset of participants from Experiment 1 (7 PCAs; 6 tADs; 14 healthy controls) took part in Experiment 2 (Table 1). Experiment 2 was conducted in the same experimental setting as Experiment 1.

##### 2.2.2.1. Environmental conditions

Participants were seated within reaching distance of the target and asked to pick up the target under the following environmental conditions:

1. Clutter: The target was presented in isolation (no distractors) or among 2 or 5 distractors (Figure 1Di).
2. Cue: The target was presented with or without the same visual color contrast cue reported in Experiment 1 (Figure 1B).
3. Target position: The target object was presented centrally (body midline) or laterally (left or right) in near or far-reachable space, for a total of six positions (Figure 1Dii).

##### 2.2.2.2. Procedure

Between each trial, the participants' view of the setting was obscured by a blind and the participants were instructed to keep their dominant hand on their lap. Trials were administered through a repeated-measures design such that each participant performed 36 trials, one for each combination of clutter (0, 2 or 5 distractor cups); cue (not present/present) and target position (1, 2, 3, 4, 5, and 6). To control for order effects cue and clutter variables were arranged in six counterbalanced variants of a Latin square design with variants randomly assigned to each participant.

### 2.3. Data collection

Completion time was defined as the time interval between the start of each trial and when the participant's hand first came in contact with the target object. An inertial measurement unit (IMU) mounted within the base of the target object recorded its movement at 75 Hz (Figure 1E). IMU threshold acceleration values were used automatically to calculate trial time based on detected target movement (automatically calculated trials: Experiment 1: 1,312/1,344 (97.8%); Experiment 2: 957/972 (98.5%)); the remainder were manually determined. A total of 12 trials were missing from Experiment 1 owing to three participants picking up a distractor rather than target object: two PCA (missing either 2 or 3 trials) and one tAD participant (missing 7 trials).

### 2.4. Statistical methods

For experiment 1, the full data analysis model was a linear mixed effects model for the log-transformed completion times. Completion times were log-transformed so that normality assumptions were not materially violated. All results were back-transformed (exponentiated) to permit interpretation of results as geometric means, ratios of geometric means (or percentage differences), and ratios of these ratios when making comparisons between two patient groups. The model was as follows.


(1) $$\begin{array}{l} \log_{e}\left(t_{ijk}\right)=\beta_{0i}+\overset{6}{\underset{h=1}{\sum }}\beta_{hi}u_{hijk}+b_{0ij}+\overset{6}{\underset{h=1}{\sum }}b_{hij}u_{hijk}+\varepsilon_{ijk} \end{array}$$

(2) $$\begin{array}{l} with\;b_{hij}\sim N\left(0,\sigma_{hi}^{2}\right)\;and\;\varepsilon_{ijk}\sim N\left(0,\sigma_{i}^{2}\right),\;all\;independently \end{array}$$

where: *t*_*ijk*_= time for the *k*th repeated measure for the *j*th participant in the *i*th group and the *u*_*hijk*_(*h*= 1 to 6) are indicator variables for the environmental conditions (number of distractors, cue, target position and starting position (the latter has four values so needs three indicators)). For example, *u*_1*ijk*_ is an indicator variable taking the values 0 and 1 according to whether the *k*th repeated measure for the *j*th participant in the *i*th group involves 2 or 5 distractors (where *h* = 1 represents the distractor variable).

The β_1*i*_ are the group-specific distractor effects; the β_2*i*_ are the group-specific cue effects; the β_3*i*_ are the group-specific target position effects; and the β_4*i*_, β_5*i*_, β_6*i*_ are the group-specific starting position effects.

The *b*_0*ij*_ are random effects that allow for associations between pairs of measurements from the same participant. The *b*_*hij*_ (*h*= 1 to 6) are random effects that allow these associations to be greater when the pair involves shared environmental conditions (for example, a shared target position). If not statistically significant (using likelihood ratio tests) or the models did not converge these latter terms were omitted. The ε_*ijk*_ are individual level residuals.

An analogous model was used for experiment 2. In neither model was there evidence to include fixed effect interactions other than those between groups and each environmental condition. For each experiment, linear contrasts of parameter estimates were used to estimate (with 95% CI) each of the following:

1. Geometric mean completion times in each group (averaging over all environmental conditions);
2. Geometric mean completion times for each environmental condition in each group (averaging over all other environmental conditions);
3. Group-specific environment effects: defined as ratios of the environment-specific geometric means in 2) (e.g., for Experiment 1, cue vs. no cue; 5 vs. 2 distractors etc.); and
4. Pairwise between-group comparisons: ratios of the group-specific environment effects in 3) (i.e., ratios of ratios).

Wald tests were used to compare geometric mean completion times, first with a joint test across all three patient groups and then by estimating pairwise group comparisons (PCA vs. Control, tAD vs. Control, and tAD vs. PCA). Similarly, for each environmental condition, tests were performed to investigate whether the effect of the environmental condition differed between groups first using a joint test across all three groups and then separately for each individual pairwise between group comparison.

All analyses were carried out in Stata v.16.

## 3. Results

### 3.1. Experiment 1: Reaching from standing positions

Averaged over all conditions, completion times were longer (*p* < 0.0001, joint test) in PCA [estimated geometric mean completion time: 5.43 sec (95%CI: 4.36, 6.77)] and tAD [4.38 sec (3.97, 4.84), compared to the control group [2.85 sec (2.64, 3.08)]. Pairwise differences between each patient group and controls were formally statistically significant (*p* < 0.001, both tests) whilst that between the two patient groups was not (*p* = 0.081).

#### 3.1.1. Environmental conditions

There was no formal statistical evidence of an effect of clutter or the color contrast cue on completion time within any of the three groups; as expected, completion time was shorter when the target was positioned nearer (Table 3A). There was no evidence that the effect of clutter or cue or target position differed between the three groups (clutter, *p* = 0.25; cue, *p* = 0.98; target position, *p* = 0.25).

**Table 3:** Experiment 1: Estimated geometric mean completion time comparisons expressed as ratios (95% CI).

**(A) Main effects of environmental conditions**			
	**Control**	**PCA**	**tAD**
Cue			
Present vs. absent	0.99 (0.97, 1.02)	0.99 (0.94, 1.05)	1.00 (0.94, 1.07)
Clutter			
5 vs. 2 Distractors	1.02 (1.00, 1.04)	1.06 (0.98, 1.13)	0.98 (0.94, 1.04)
Target position			
Near vs. far	0.93 (0.91, 0.95)	0.97 (0.92, 1.02)	0.91 (0.86, 0.96)
Start position			
Left vs. proximal	2.91 (2.75, 3.08)	1.94 (1.76, 2.14)	2.37 (2.15, 2.61)
Central vs. proximal	2.53 (2.39, 2.67)	1.64 (1.48, 1.80)	1.97 (1.79, 2.17)
Right vs. proximal	2.91 (2.75, 3.08)	1.92 (1.74, 2.11)	2.30 (2.08, 2.53)
**(B) Environmental conditions by group interactions**			
	**PCA vs. controls**	**tAD vs. controls**	**tAD vs. PCA**
Cue			
Present vs. absent	1.00 (0.94, 1.06)	1.01 (0.94, 1.08)	1.01 (0.93, 1.10)
Clutter			
5 vs. 2 distractors	1.04 (0.96, 1.12)	0.97 (0.91, 1.02)	0.93 (0.85, 1.02)
Target position			
Near vs. far	1.04 (0.98, 1.10)	0.98 (0.92, 1.03)	0.94 (0.87, 1.01)
Start position			
Left vs. proximal	0.67 (0.60, 0.75)	0.82 (0.73, 0.91)	1.22 (1.07, 1.40)
Central vs. proximal	0.65 (0.58, 0.72)	0.78 (0.70, 0.87)	1.21 (1.05, 1.38)
Right vs. proximal	0.66 (0.59, 0.74)	0.79 (0.70, 0.88)	1.20 (1.04, 1.38)

Estimated geometric mean completion times expressed as (A) between condition ratios comparing cue, clutter, target and start position conditions within each participant group, and (B) ratios comparing cue, clutter, target, and start position ratios between participant groups. 95% CIs are in brackets; PCA, posterior cortical atrophy; tAD, typical Alzheimer's disease.

Expectedly, completion times in all groups were longer when reaching to the target object under distant relative to proximal (within reaching distance of the target) starting standing positions. However, distant vs. proximal ratios of completion times were greater in controls than in either patient group. For distant vs. proximal starting positions, estimated ratios of completion times were between 2.53 and 2.91 (i.e., between 153% and 191% increases) in the control group, but between 1.64 and 1.94 (64% to 94% increases) in the PCA group, with the corresponding ratios of these between route ratios for PCA vs. controls being 0.65 [1.64/2.53 = 0.65; 95%CI (0.58, 0.72)] and 0.67 [1.94/2.91 = 0.67; 95%CI (0.60, 0.75)] (Table 3B). Estimated ratios of completion times between distant vs. proximal starting positions in the tAD group were intermediate between those for the PCA and control groups.

Formal tests of differences between interaction terms provided evidence that the effect of starting position differed between controls and both patient groups (vs. PCA: *p* < 0.0001; vs. tAD: *p* < 0.0001) and between patient groups (*p* = 0.012). Relative to controls, PCA and to a lesser extent tAD groups were particularly inefficient at locating the target under proximal compared to distant starting standing positions.

### 3.2. Experiment 2: Reaching from a seated position

A subset of participants conducted the same task from a fixed seated position comparable to the proximal starting position under a greater number of clutter and target position conditions (Experiment 2). Averaged over all conditions, completion times were longer in PCA geometric mean completion time: 2.05 sec [95% CI 1.55, 2.70] and tAD [1.96 sec (1.63, 2.36)], compared to control groups [1.27 sec (1.15, 1.41)]. Pairwise differences between each patient group and controls were formally statistically significant (*p* ≤ 0.002, both tests) whilst that between the two patient groups was not (*p* = 0.804).

#### 3.2.1. Environmental conditions

**Figure 2:**
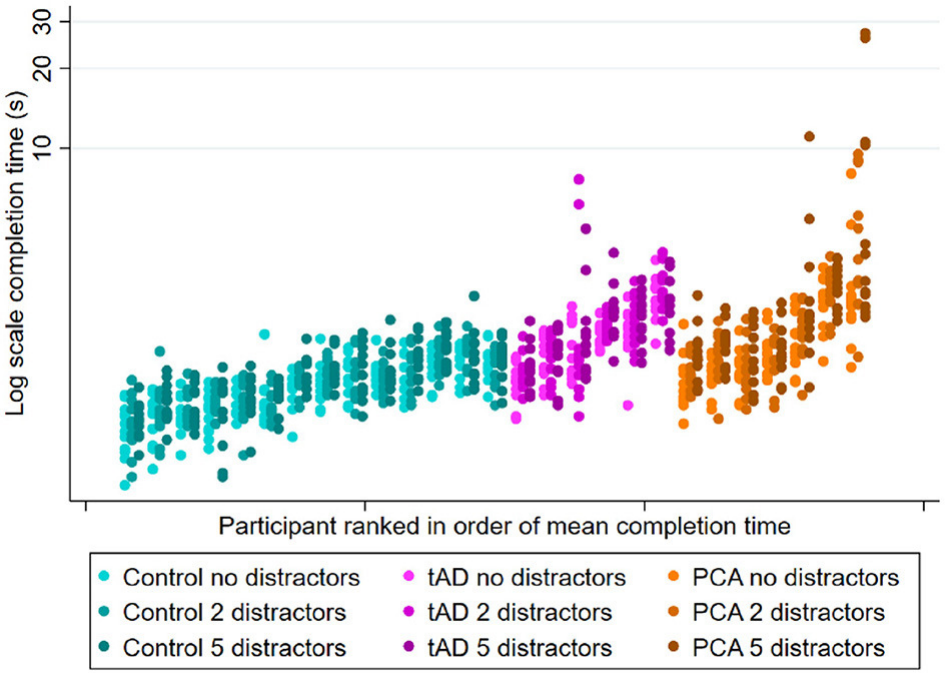
Experiment 2 scatter plots of observed completion time for each participant group under different clutter conditions (no, 2, and 5 distractors). Plots show individual completion times per participant per trial. Patients within each group are ranked left to right in order of mean completion time averaging over all conditions.

See Figure 2 for observed completion times for each participant under different clutter conditions. An effect of clutter on completion time was observed within all three groups, with completion times being longer when reaching for the target object surrounded by distractors relative to being presented in isolation (Table 4A). However, having five distractors rather than none was associated with a disproportionately greater effect in PCA patients than in controls or tAD groups, with geometric mean completion times being 29% longer (ratio = 1.29) in PCA patients but only 9% longer (ratio = 1.09) in both controls and tAD. This represented an additional relative increase of 18% [1.29/1.09 = 1.18; 95%CI (1.06, 1.33)] for PCA vs. Control, and a relative decrease of 16% [1.09/1.29 = 0.84; 95%CI (0.74, 0.96)] for tAD vs PCA (Table 4B). Having two distractors rather than none produced smaller percentage increases in completion times for each group; and smaller, not statistically significant but directionally consistent, ratios of relative effects: a relative increase of 8% [1.12/1.04 = 1.08; 95%CI (0.96, 1.21)] for PCA vs. Control, and a relative decrease of 5% [1.06/1.12 = 0.94; 95%CI (0.83, 1.08)] for tAD vs. PCA.

**Table 4:** Experiment 2: Estimated geometric mean completion time comparisons expressed as ratios (95% CI).

**(A) Main effects of environmental conditions**			
	**Control**	**PCA**	**tAD**
Cue			
Present vs. absent	1.01 (0.99, 1.04)	1.03 (0.95, 1.11)	1.06 (0.97, 1.17)
Clutter			
2 vs. 0 distractors	1.04 (1.01, 1.07)	1.12 (1.00, 1.25)	1.06 (0.99, 1.13)
5 vs. 0 distractors	1.09 (1.06, 1.12)	1.29 (1.16, 1.44)	1.09 (1.01, 1.17)
Target position			
Position 1 vs. 2	1.09 (1.04, 1.13)	1.17 (0.96, 1.41)	1.10 (0.97, 1.26)
Position 3 vs. 2	1.06 (1.01, 1.10)	1.16 (0.96, 1.40)	1.08 (0.94, 1.23)
Position 4 vs. 2	0.90 (0.86, 0.94)	1.19 (0.98, 1.44)	0.92 (0.81, 1.05)
Position 5 vs. 2	0.80 (0.77, 0.84)	0.90 (0.74, 1.09)	0.83 (0.72, 0.94)
Position 6 vs. 2	0.88 (0.84, 0.92)	1.06 (0.88, 1.29)	0.83 (0.73, 0.94)
**(B) Environmental conditions by group interactions**			
	**PCA vs. Control**	**tAD vs. Control**	**tAD vs. PCA**
Cue			
Present vs. absent	1.02 (0.94, 1.10)	1.05 (0.95, 1.16)	1.03 (0.92, 1.16)
Clutter			
2 vs. 0 distractors	1.08 (0.96, 1.21)	1.02 (0.94, 1.10)	0.94 (0.83, 1.08)
5 vs. 0 distractors	1.18 (1.06, 1.33)	1.00 (0.92, 1.08)	0.84 (0.74, 0.96)
Target Position			
Position 1 vs. 2	1.07 (0.88, 1.31)	1.01 (0.88, 1.16)	0.94 (0.75, 1.19)
Position 3 vs. 2	1.09 (0.9, 1.33)	1.02 (0.89, 1.17)	0.93 (0.74, 1.17)
Position 4 vs. 2	1.33 (1.09, 1.62)	1.03 (0.90, 1.18)	0.78 (0.61, 0.98)
Position 5 vs. 2	1.12 (0.92, 1.36)	1.03 (0.90, 1.18)	0.92 (0.73, 1.16)
Position 6 vs. 2	1.21 (0.99, 1.47)	0.94 (0.82, 1.08)	0.78 (0.62, 0.98)

Estimated geometric mean completion times expressed as (A) between condition ratios comparing cue, clutter and target position conditions within each participant group, and (B) ratios comparing cue, clutter, and target position ratios between participant groups. 95% CIs are in brackets. PCA, posterior cortical atrophy; tAD, typical Alzheimer's disease.

Formal tests of interaction provided evidence that the effect of clutter differed between PCA and other participant groups (vs. Control: *p* = 0.015; vs tAD: *p* = 0.031), but not between tAD and control groups (*p* = 0.84). As in Experiment 1, there was no evidence of an effect of the color contrast cue on completion time within any of the three groups (Table 4A).

While estimated ratios of completion times were lower for targets under positions 4 and 6 in the control group (0.90 and 0.88, respectively), there was a non-statistically significant tendency toward corresponding ratios being higher for the PCA group (1.19 and 1.06). Overall tests of interaction found that the effect of target position was not formally statistically significantly different between PCA and other participant groups (vs. Control: *p* = 0.081; vs. tAD: *p* = 0.15).

## 4. Discussion

The current investigation evaluated effects of environmental conditions on object localization in PCA and tAD within a controlled setting. Overall, both patient groups took longer to locate a target object than healthy controls across two experiments. All participants completed the task from starting standing positions at varying distances from the target (Experiment 1), a subset subsequently completed the task from a fixed seated position (Experiment 2). In Experiment 1, there was no evidence of an effect of visual clutter or the presence of a color contrast cue on performance within any participant group. Similarly to Experiment 1, in Experiment 2 there was no evidence of an effect of the color contrast cue on performance within any participant group. However, not only did all three groups take longer to reach the target object when it was presented among visual clutter compared to being presented in isolation, there was also evidence that the effect of visual clutter on completion time was greater in PCA relative to both control and tAD groups. Effects of clutter on aspects of functional independence relating to clinical phenotype (visual-led more so than memory-led) may carry implications for tailoring environmental adaptations based on symptom profile.

The apparent inconsistency in effects of clutter across Experiments 1 and 2 may have related to differences in experimental conditions: Experiment 2 included trials where the object was presented in isolation, featured more target positions and was conducted at a fixed, proximal distance to targets from a seated position. Overall, findings are consistent with documented effects of clutter in neurodegenerative syndromes (1) and emphasize the impact of reducing surrounding visual clutter on reaching function in PCA (19, 44, 45).

The lack of the cue effect may have related to a number of visual deficits, such as excessive visual crowding in PCA resulting in difficulty perceiving the target when flanked by additional visual features introduced by the cue (19, 46). An anticipated benefit of the visual cue was to increase target visual saliency following documented effects of conspicuous, visually salient parts of scenes (for example, relating to variation in color, intensity and orientation) on visual search efficiency in PCA and to a lesser extent tAD (47). However, it is possible that introducing the color contrast cue did not materially increase visual saliency of the target relative to surroundings, given the target and distractors themselves differed in color (Figure 1).

In Experiment 1, while all three groups—as expected—performed more efficiently when standing in front of the table compared to approaching it from further away, overall healthy controls incurred a greater penalty in completion time when traveling a greater distance. In Experiment 2, all groups were faster at localizing the target object when it was presented centrally and in close proximity, as expected. There was an observed (non-statistically significant) tendency for the advantage in locating objects in close proximity to be diminished in PCA patients. This may reflect previously reported restrictions in the effective visual field in PCA, particularly limiting localization of objects which despite being in close proximity are also positioned in peripheral vision and the inferior visual field (48, 49). However, there was no evidence that effects of target position differed between groups. Moreover, it is possible that varying cues about self-motion from optic flow may contribute to aspects of performance, including particular difficulty locating the target from proximal vs. distant starting positions (Experiment 1). While case studies of PCA suggest better localization of moving relative to static objects (50, 51), PCA group studies have suggested impaired discrimination of optic flow (52) and/or have not provided evidence of visual motion cues on navigation (26). Future work might clarify the contribution of visual motion cues on object localization.

The current investigations had a number of limitations. Firstly, while study strengths include the number of observations per participant, findings are from a small and heterogenous group of mostly young-onset patients and should be replicated and validated in larger samples. Secondly, conducting the tasks within a controlled experimental setting may limit how much the findings can be generalized to other settings. And thirdly, the study did not formally investigate the processes that may underly object localization behaviors in PCA and tAD. To further disentangle the mechanisms that give rise to the object localization deficits in PCA and tAD described in this study, future investigations may benefit from investigating eye and body motion tracking and assessing whether the impact of clutter depends on how similar/dissimilar it is in shape and color compared to the target.

The present study provides modest evidence of environmental conditions influencing efficiency in interacting with real-world objects. Findings underscore the impact of dementia-related visual loss on functional status, and highlight the importance of considering dementia diagnosis along with task and environmental conditions to inform approaches supporting patients to engage in everyday activities independently, including those involving object localization skills in PCA. Such interventions may include ensuring that target objects are presented with a limited number of distractors. Decreasing the proximity of target and distractors and the functional and visual similarity between the two may offer further benefits (19, 20).

## Data availability statement

The raw data supporting the conclusions of this article will be made available by the authors, without undue reservation.

## Ethics statement

The studies involving human participants were reviewed and approved by National Research Ethics Service Committee London Queen Square. The patients/participants provided their written informed consent to participate in this study.

## Author contributions

DO took the lead in writing the manuscript with SP, TS, TP, IM, SC, CF, and KY providing critical feedback and helping to shape the manuscript and provided support for data analysis by TP and CF. KY recruited the study participants, carried out the experiments with the support of IM, TS, and SP, and provided support for data analysis by TP and CF and the writeup of the manuscript. SC conceived and planned the experiment with the support of NT and KY. All authors contributed to the article and approved the submitted version.
